# (*E*)-3-(3-Chloro­phen­yl)-1-(4-methoxy­phen­yl)prop-2-en-1-one

**DOI:** 10.1107/S1600536810017150

**Published:** 2010-05-15

**Authors:** Naveed Ahmad, Hamid Latif Siddiqui, Muhammad Zia-ur-Rehman, Masood Parvez

**Affiliations:** a Institute of Chemistry, University of the Punjab, Lahore 54590, Pakistan; b Applied Chemistry Research Centre, PCSIR Laboratories Complex, Lahore-54600, Pakistan; c Department of Chemistry, The University of Calgary, 2500 University Drive NW, Calgary, Alberta, Canada T2N 1N4

## Abstract

The title mol­ecule, C_16_H_13_ClO_2_, is *trans* with respect to the C=C double bond. The dihedral angles between the mean plane of the prop-2-en-1-one unit and those of the 3-chloro- and 4-meth­oxy-substituted benzene rings are 20.93 (9) and 20.42 (10)°, respectively, and the dihedral angle between the mean planes of the two benzene rings is 40.96 (5)°. The crystal structure is stabilized by weak inter­molecular C—H⋯O hydrogen bonds, forming chains along the *b* axis.

## Related literature

For the biological activity of chalcones, see: Dimmock *et al.* (1999 ▶); Opletalova & Sedivy (1999 ▶); Lin *et al.* (2002 ▶); Nowakowska (2007 ▶). For the synthesis and biological activity of related chalcone derivatives, see: Hussain *et al.* (2009 ▶). For non-linear optical studies of chalcones, see: Sarojini *et al.* (2006 ▶); Poornesh *et al.* (2009 ▶); Shettigar *et al.* (2006 ▶; 2008 ▶). For related structures, see: Rosli *et al.* (2006 ▶); Patil *et al.* (2006 ▶); Harrison *et al.* (2006 ▶); Fun *et al.* (2008 ▶); Jasinski *et al.* (2010 ▶).
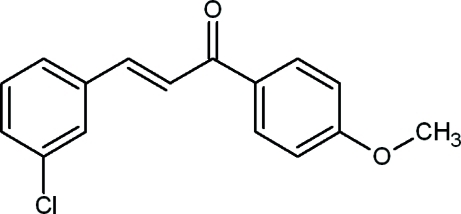


## Experimental

### 

#### Crystal data

C_16_H_13_ClO_2_
*M* *_r_* = 272.71
Monoclinic, 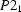

*a* = 10.3415 (6) Å
*b* = 3.8938 (1) Å
*c* = 16.9152 (10) Å
β = 107.582 (2)°
*V* = 649.32 (6) Å^3^
*Z* = 2
Mo *K*α radiation
μ = 0.29 mm^−1^
*T* = 173 K
0.18 × 0.16 × 0.04 mm

#### Data collection

Nonius KappaCCD diffractometer
Absorption correction: multi-scan (*SORTAV*; Blessing, 1997 ▶) *T*_min_ = 0.950, *T*_max_ = 0.989
2099 measured reflections
2099 independent reflections
2075 reflections with *I* > 2σ(*I*)

#### Refinement

*R*[*F* ^2^ > 2σ(*F* ^2^)] = 0.026
*wR*(*F* ^2^) = 0.073
*S* = 1.15
2099 reflections
173 parameters
1 restraint
H-atom parameters constrained
Δρ_max_ = 0.13 e Å^−3^
Δρ_min_ = −0.14 e Å^−3^
Absolute structure: Flack (1983 ▶), 687 Friedel pairs
Flack parameter: 0.08 (6)

### 

Data collection: *COLLECT* (Hooft, 1998 ▶); cell refinement: *DENZO* (Otwinowski & Minor, 1997 ▶); data reduction: *SCALEPACK* (Otwinowski & Minor, 1997 ▶); program(s) used to solve structure: *SHELXS97* (Sheldrick, 2008 ▶); program(s) used to refine structure: *SHELXL97* (Sheldrick, 2008 ▶); molecular graphics: *ORTEP-3 for Windows* (Farrugia, 1997 ▶); software used to prepare material for publication: *SHELXL97*.

## Supplementary Material

Crystal structure: contains datablocks global, I. DOI: 10.1107/S1600536810017150/lh5044sup1.cif

Structure factors: contains datablocks I. DOI: 10.1107/S1600536810017150/lh5044Isup2.hkl

Additional supplementary materials:  crystallographic information; 3D view; checkCIF report

## Figures and Tables

**Table 1:** Hydrogen-bond geometry (Å, °)

*D*—H⋯*A*	*D*—H	H⋯*A*	*D*⋯*A*	*D*—H⋯*A*
C7—H7*A*⋯O2^i^	0.98	2.58	3.545 (2)	168
C16—H16⋯O1^ii^	0.95	2.51	3.424 (2)	162

Symmetry codes: (i) 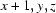; (ii) 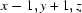.

**Fig. 1.**
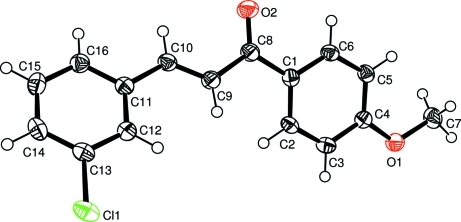
The title molecule plotted with the displacement ellipsoids at 50% probability level (Farrugia, 1997).

**Fig. 2.**
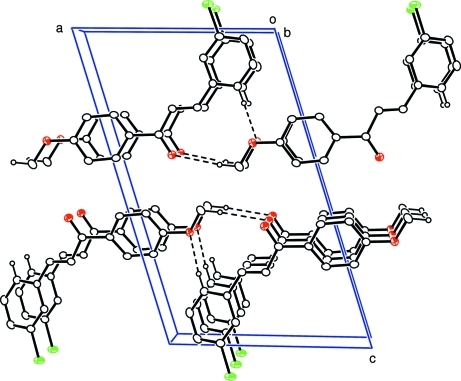
Unit cell packing of the title molecule, viewed down the b-axis. Intramolecular interactions of the type C—H···O are shown as dashed lines and H-atoms not involved in hydrogen bonding interactions have been excluded.

## supplementary crystallographic information

### Comment

Chalcones are well known for their biological activities (Dimmock *et al.*, 1999). These have been reported as potential anti-fungal chemotherapeutic (Opletalova & Sedivy, 1999), anti-tuberculosis (Lin *et al.*, 2002) and anti-infective & anti-inflammatory agents (Nowakowska, 2007). In addition, few among these have found their use as organic non-linear optical materials (NLO) due to their good SHG (second-harmonic generation) conversion efficiencies (Sarojini *et al.*, 2006; Poornesh *et al.*, 2009; Shettigar *et al.*, 2006; 2008). In continuation of our work on chalcones (Hussain *et al.*, 2009) and in view of the importance of chloro chalcones, the synthesis and crystal structure of the title compound, (I), is presented in this article.

The title molecule (Fig. 1) exhibits an *E* configuration with respect to the C═C double bond, the torsion angle C–C═C–C being -177.75 (17)°. The dihedral angle between the mean planes of the 3-chloro and 4-methoxy substituted benzene rings is 40.96 (5)°. The dihedral angles between the mean planes of the prop-2-en-1-one unit and those of the 3-chloro and 4-methoxy substitued benzene rings are 20.93 (9) and 20.42 (10)°, respectively. The geometrical parameters for (I) are consistent with those of some recently reported chalcone derivatives closely related to (I) (Rosli *et al.*, 2006; Patil *et al.*, 2006; Harrison *et al.*, 2006; Fun *et al.*; 2008; Jasinski *et al.*, 2010). The structure is stabilized by intermolecular interactions of the type C—H···O resulting in polymeric chains along the *b*-axis (Fig. 2, Tab. 1)

### Experimental

A mixture of 3-chlorobenzaldehyde (0.01 moles, 1.13 g), 4-methoxyacetophenone (0.01 moles, 1.37 ml) and sodium hydroxide solution (10%, 30 ml) was stirred at room temperature for 6 hrs. Precipitates obtained were poured into ice-cold water (500 ml) and left to stand for 2 hours followed by filtration of the resultant solid which was dried and crystallized from ethanol by slow evaporation.

### Refinement

The H-atoms were clocated from difference Fourier maps and were included in the refinement at geometrically idealized positions in riding-model approximation with C—H = 0.95 and 0.98 Å for aryl and methyl type H-atoms, respectively; the *U*_iso_(H) were allowed at 1.2*U*_eq_(C). The final difference map was essentially featurless.

### Crystal data

**Table:**

C_16_H_13_ClO_2_	*F*(000) = 284
*M**_r_* = 272.71	*D*_x_ = 1.395 Mg m^−^^3^
Monoclinic, *P*2_1_	Mo *K*α radiation, λ = 0.71073 Å
Hall symbol: P 2yb	Cell parameters from 1186 reflections
*a* = 10.3415 (6) Å	θ = 1.0–27.5°
*b* = 3.8938 (1) Å	µ = 0.29 mm^−^^1^
*c* = 16.9152 (10) Å	*T* = 173 K
β = 107.582 (2)°	Plate, colourless
*V* = 649.32 (6) Å^3^	0.18 × 0.16 × 0.04 mm
*Z* = 2	

### Data collection

**Table:**

Nonius KappaCCD diffractometer	2099 independent reflections
Radiation source: fine-focus sealed tube	2075 reflections with *I* > 2σ(*I*)
graphite	*R*_int_ = 0.000
ω and φ scans	θ_max_ = 25.0°, θ_min_ = 2.1°
Absorption correction: multi-scan (*SORTAV*; Blessing, 1997)	*h* = −12→12
*T*_min_ = 0.950, *T*_max_ = 0.989	*k* = −4→4
2099 measured reflections	*l* = −20→19

### Refinement

**Table:**

Refinement on *F*^2^	Secondary atom site location: difference Fourier map
Least-squares matrix: full	Hydrogen site location: difference Fourier map
*R*[*F*^2^ > 2σ(*F*^2^)] = 0.026	H-atom parameters constrained
*wR*(*F*^2^) = 0.073	*w* = 1/[σ^2^(*F*_o_^2^) + (0.0313*P*)^2^ + 0.151*P*] where *P* = (*F*_o_^2^ + 2*F*_c_^2^)/3
*S* = 1.15	(Δ/σ)_max_ < 0.001
2099 reflections	Δρ_max_ = 0.13 e Å^−^^3^
173 parameters	Δρ_min_ = −0.14 e Å^−^^3^
1 restraint	Absolute structure: Flack (1983), 687 Friedel pairs
Primary atom site location: structure-invariant direct methods	Flack parameter: 0.08 (6)

### Special details

**Table:**

Geometry. All esds (except the esd in the dihedral angle between two l.s. planes) are estimated using the full covariance matrix. The cell esds are taken into account individually in the estimation of esds in distances, angles and torsion angles; correlations between esds in cell parameters are only used when they are defined by crystal symmetry. An approximate (isotropic) treatment of cell esds is used for estimating esds involving l.s. planes.
Refinement. Refinement of *F*^2^ against ALL reflections. The weighted *R*-factor wR and goodness of fit *S* are based on *F*^2^, conventional *R*-factors *R* are based on *F*, with *F* set to zero for negative *F*^2^. The threshold expression of *F*^2^ > σ(*F*^2^) is used only for calculating *R*-factors(gt) etc. and is not relevant to the choice of reflections for refinement. *R*-factors based on *F*^2^ are statistically about twice as large as those based on *F*, and *R*- factors based on ALL data will be even larger.

### Fractional atomic coordinates and isotropic or equivalent isotropic displacement parameters (Å^2^)

**Table:**

	*x*	*y*	*z*	*U*_iso_*/*U*_eq_	
Cl1	0.28612 (5)	0.84356 (13)	−0.07337 (2)	0.03991 (15)	
O1	1.26882 (11)	0.5880 (4)	0.35589 (7)	0.0330 (3)	
O2	0.67938 (12)	0.6392 (4)	0.39544 (8)	0.0413 (4)	
C1	0.85889 (16)	0.6328 (4)	0.33613 (10)	0.0247 (4)	
C2	0.90929 (17)	0.7521 (4)	0.27311 (10)	0.0267 (4)	
H2	0.8490	0.8486	0.2242	0.032*	
C3	1.04622 (18)	0.7304 (5)	0.28148 (10)	0.0290 (4)	
H3	1.0797	0.8125	0.2385	0.035*	
C4	1.13507 (16)	0.5884 (4)	0.35290 (10)	0.0253 (4)	
C5	1.08649 (17)	0.4606 (5)	0.41555 (10)	0.0264 (4)	
H5	1.1465	0.3585	0.4637	0.032*	
C6	0.94934 (17)	0.4851 (5)	0.40622 (10)	0.0261 (4)	
H6	0.9158	0.3989	0.4488	0.031*	
C7	1.36446 (17)	0.4448 (5)	0.42797 (12)	0.0365 (5)	
H7A	1.4561	0.4645	0.4229	0.044*	
H7B	1.3430	0.2022	0.4330	0.044*	
H7C	1.3598	0.5697	0.4773	0.044*	
C8	0.71475 (17)	0.6756 (5)	0.33309 (10)	0.0287 (4)	
C9	0.61392 (17)	0.7636 (5)	0.25241 (10)	0.0284 (4)	
H9	0.6357	0.7207	0.2026	0.034*	
C10	0.49465 (16)	0.9000 (5)	0.24782 (10)	0.0276 (4)	
H10	0.4780	0.9468	0.2990	0.033*	
C11	0.38516 (17)	0.9868 (5)	0.17214 (11)	0.0252 (3)	
C12	0.38969 (16)	0.8919 (5)	0.09297 (10)	0.0267 (4)	
H12	0.4662	0.7735	0.0865	0.032*	
C13	0.28167 (17)	0.9727 (5)	0.02468 (10)	0.0280 (4)	
C14	0.16803 (17)	1.1442 (5)	0.03152 (11)	0.0311 (4)	
H14	0.0946	1.1960	−0.0164	0.037*	
C15	0.16385 (18)	1.2383 (5)	0.10952 (12)	0.0325 (4)	
H15	0.0870	1.3567	0.1154	0.039*	
C16	0.27161 (17)	1.1606 (5)	0.17948 (11)	0.0290 (4)	
H16	0.2677	1.2268	0.2328	0.035*	

### Atomic displacement parameters (Å^2^)

**Table:**

	*U*^11^	*U*^22^	*U*^33^	*U*^12^	*U*^13^	*U*^23^
Cl1	0.0526 (3)	0.0409 (3)	0.0234 (2)	−0.0034 (2)	0.00716 (18)	0.0004 (2)
O1	0.0243 (6)	0.0444 (8)	0.0294 (6)	0.0004 (6)	0.0068 (5)	0.0020 (6)
O2	0.0302 (6)	0.0675 (10)	0.0268 (6)	0.0045 (7)	0.0093 (5)	0.0055 (7)
C1	0.0258 (8)	0.0247 (8)	0.0214 (8)	−0.0002 (7)	0.0037 (6)	−0.0034 (7)
C2	0.0300 (9)	0.0279 (9)	0.0201 (8)	0.0004 (7)	0.0044 (6)	0.0020 (7)
C3	0.0323 (8)	0.0330 (9)	0.0217 (8)	−0.0033 (7)	0.0082 (6)	−0.0001 (7)
C4	0.0255 (8)	0.0254 (8)	0.0242 (8)	−0.0018 (7)	0.0061 (6)	−0.0042 (7)
C5	0.0280 (8)	0.0268 (8)	0.0216 (8)	0.0014 (7)	0.0034 (6)	−0.0002 (7)
C6	0.0313 (8)	0.0264 (8)	0.0207 (8)	−0.0007 (7)	0.0080 (6)	−0.0009 (7)
C7	0.0260 (8)	0.0436 (12)	0.0349 (10)	0.0017 (8)	0.0017 (7)	0.0037 (9)
C8	0.0284 (8)	0.0325 (9)	0.0235 (8)	−0.0005 (8)	0.0055 (7)	−0.0017 (8)
C9	0.0262 (8)	0.0349 (10)	0.0227 (8)	−0.0006 (7)	0.0053 (6)	−0.0029 (8)
C10	0.0294 (8)	0.0307 (10)	0.0222 (8)	−0.0002 (8)	0.0071 (6)	0.0001 (7)
C11	0.0244 (7)	0.0245 (8)	0.0263 (8)	−0.0041 (7)	0.0069 (6)	0.0010 (7)
C12	0.0259 (8)	0.0266 (9)	0.0270 (8)	−0.0022 (7)	0.0070 (6)	−0.0001 (8)
C13	0.0331 (9)	0.0246 (8)	0.0250 (8)	−0.0075 (7)	0.0067 (7)	0.0008 (7)
C14	0.0276 (8)	0.0280 (9)	0.0319 (9)	−0.0034 (8)	0.0003 (7)	0.0064 (8)
C15	0.0249 (8)	0.0292 (10)	0.0427 (10)	0.0014 (7)	0.0090 (7)	0.0026 (8)
C16	0.0284 (8)	0.0298 (9)	0.0291 (9)	−0.0014 (8)	0.0091 (7)	−0.0001 (8)

### Geometric parameters (Å, °)

**Table:**

Cl1—C13	1.747 (2)	C7—H7C	0.9800
O1—C4	1.369 (2)	C8—C9	1.486 (2)
O1—C7	1.431 (2)	C9—C10	1.323 (2)
O2—C8	1.225 (2)	C9—H9	0.9500
C1—C6	1.393 (2)	C10—C11	1.470 (2)
C1—C2	1.400 (2)	C10—H10	0.9500
C1—C8	1.485 (2)	C11—C16	1.393 (2)
C2—C3	1.383 (2)	C11—C12	1.404 (2)
C2—H2	0.9500	C12—C13	1.379 (2)
C3—C4	1.393 (2)	C12—H12	0.9500
C3—H3	0.9500	C13—C14	1.387 (3)
C4—C5	1.395 (2)	C14—C15	1.383 (3)
C5—C6	1.382 (2)	C14—H14	0.9500
C5—H5	0.9500	C15—C16	1.392 (2)
C6—H6	0.9500	C15—H15	0.9500
C7—H7A	0.9800	C16—H16	0.9500
C7—H7B	0.9800		
			
C4—O1—C7	117.56 (13)	O2—C8—C9	120.56 (15)
C6—C1—C2	118.44 (15)	C1—C8—C9	118.44 (14)
C6—C1—C8	118.99 (14)	C10—C9—C8	121.97 (15)
C2—C1—C8	122.48 (14)	C10—C9—H9	119.0
C3—C2—C1	120.48 (15)	C8—C9—H9	119.0
C3—C2—H2	119.8	C9—C10—C11	127.06 (15)
C1—C2—H2	119.8	C9—C10—H10	116.5
C2—C3—C4	120.00 (15)	C11—C10—H10	116.5
C2—C3—H3	120.0	C16—C11—C12	119.01 (16)
C4—C3—H3	120.0	C16—C11—C10	118.85 (15)
O1—C4—C3	115.28 (14)	C12—C11—C10	122.11 (15)
O1—C4—C5	124.30 (15)	C13—C12—C11	119.15 (16)
C3—C4—C5	120.42 (15)	C13—C12—H12	120.4
C6—C5—C4	118.77 (15)	C11—C12—H12	120.4
C6—C5—H5	120.6	C12—C13—C14	122.17 (16)
C4—C5—H5	120.6	C12—C13—Cl1	118.86 (14)
C5—C6—C1	121.85 (15)	C14—C13—Cl1	118.94 (13)
C5—C6—H6	119.1	C15—C14—C13	118.62 (15)
C1—C6—H6	119.1	C15—C14—H14	120.7
O1—C7—H7A	109.5	C13—C14—H14	120.7
O1—C7—H7B	109.5	C14—C15—C16	120.40 (16)
H7A—C7—H7B	109.5	C14—C15—H15	119.8
O1—C7—H7C	109.5	C16—C15—H15	119.8
H7A—C7—H7C	109.5	C15—C16—C11	120.65 (16)
H7B—C7—H7C	109.5	C15—C16—H16	119.7
O2—C8—C1	120.99 (15)	C11—C16—H16	119.7
			
C6—C1—C2—C3	−1.5 (3)	O2—C8—C9—C10	19.7 (3)
C8—C1—C2—C3	174.97 (16)	C1—C8—C9—C10	−160.40 (17)
C1—C2—C3—C4	0.2 (3)	C8—C9—C10—C11	−177.75 (17)
C7—O1—C4—C3	−179.98 (16)	C9—C10—C11—C16	−173.68 (18)
C7—O1—C4—C5	0.4 (2)	C9—C10—C11—C12	8.1 (3)
C2—C3—C4—O1	−178.27 (15)	C16—C11—C12—C13	−0.1 (3)
C2—C3—C4—C5	1.4 (3)	C10—C11—C12—C13	178.09 (16)
O1—C4—C5—C6	178.08 (17)	C11—C12—C13—C14	−0.2 (3)
C3—C4—C5—C6	−1.5 (2)	C11—C12—C13—Cl1	−178.31 (13)
C4—C5—C6—C1	0.2 (3)	C12—C13—C14—C15	0.4 (3)
C2—C1—C6—C5	1.4 (3)	Cl1—C13—C14—C15	178.47 (14)
C8—C1—C6—C5	−175.25 (16)	C13—C14—C15—C16	−0.2 (3)
C6—C1—C8—O2	12.6 (3)	C14—C15—C16—C11	−0.1 (3)
C2—C1—C8—O2	−163.87 (19)	C12—C11—C16—C15	0.2 (3)
C6—C1—C8—C9	−167.35 (17)	C10—C11—C16—C15	−178.02 (16)
C2—C1—C8—C9	16.2 (2)		

### Hydrogen-bond geometry (Å, °)

**Table:**

*D*—H···*A*	*D*—H	H···*A*	*D*···*A*	*D*—H···*A*
C7—H7A···O2^i^	0.98	2.58	3.545 (2)	168
C16—H16···O1^ii^	0.95	2.51	3.424 (2)	162

Symmetry codes: (i) *x*+1, *y*, *z*; (ii) *x*−1, *y*+1, *z*.
